# An old but still valuable technique for popliteal artery stenosis: Endarterectomy via the posterior approach

**DOI:** 10.1097/MD.0000000000038693

**Published:** 2024-06-28

**Authors:** Serhat Huseyin, Orkut Guclu, Adem Reyhancan, Volkan Yuksel, Selami Gurkan, Suat Canbaz

**Affiliations:** a Cardiovascular Surgery Department, Faculty of Medicine, Trakya University, Edirne, Turkey; b Cardiovascular Surgery Department, Faculty of Medicine, Namık Kemal University, Tekirdag, Turkey.

**Keywords:** patch plasty, popliteal artery, popliteal endarterectomy, posterior approach

## Abstract

Isolated popliteal artery occlusions are rare compared with femoropopliteal occlusive diseases. Although endovascular procedures have gained importance in treatment, conventional surgery remains the gold standard. In this study, we reviewed popliteal endarterectomy and patch plasty using a posterior approach. Fourteen patients who underwent surgery for isolated popliteal artery occlusions were retrospectively examined. Patients were assessed in terms of age, sex, and risk factors, such as accompanying diseases and smoking, surgical method and anesthesia, incision type, preoperative and postoperative pulse examination, ankle-brachial indices, patency, wound infection, postoperative complications, and the treatment applied. Twelve (85.7%) patients were male, and 2 (14.3%) were female. Limb ischemia was critical (ABI < 0.7) in 11 (78.5%) patients. The average duration of postoperative hospitalization was 8 ± 3.7 days on average, and the average length of follow-up was 17 ± 3.4 months. Thrombosis and complications requiring secondary intervention did not develop during the early postoperative period. While the patency rate in the first 6 months of follow-up was 100%, it was 92.8% in the 1st year and 85.7% in the 2nd year. Surgical treatment with the posterior approach in isolated popliteal artery lesions is preferred by vascular surgeons as a prioritized treatment method, with a sufficient recanalization rate and low perioperative morbidity and mortality rates. Furthermore, it is promising because it does not prevent below-knee femoropopliteal bypass, which is the subsequent stage of treatment. Moreover, the great saphenous vein was protected, and the acceptable early- and mid-term results were encouraging.

## 1. Introduction

Peripheral artery disease (PAD) is commonly seen in the femoral region. Isolated popliteal artery occlusion is a common disease, especially in elderly patients, smokers, people with diabetes and other cardiovascular diseases.^[1]^ In addition, the pathological processes that lead to it cause morbidity and mortality by reducing or completely blocking the blood supply through the popliteal artery to the lower leg and foot. Due to tissue ischemia, these patients have significantly reduced ambulatory functioning, ability to function daily, and quality of life. Lower extremity ischemia can manifest as limping, pain at rest, or tissue loss (gangrene) and lead to limb loss.^[2]^ When part of the lower limb becomes necrotic, the patient is at risk of losing the limb and dying. Diagnosis of popliteal artery occlusive disease is important because of the risk of limb-threatening chronic ischemia.

Treatment of this disease is based on medical, interventional (endovascular), surgical, and combined methods, which have recently become popular. If some surgeons favor an interventional approach to these lesions due to technological developments in peripheral interventions, others also favor open surgery due to the structure and location of the arteries, and this topic remains controversial. The number and extent of lesions, their location, comorbidities, and short- and long-term expectations play an important role in choosing which method to use.

In the present study, we reviewed the early and mid-term follow-up results of popliteal endarterectomy + patch plasty applications with the posterior approach, which is one of the surgical treatment methods used due to atherosclerosis-related isolated popliteal artery occlusion in light of the current literature.

## 2. Method

### 2.1. Patient selection and study protocol

**Figure 1.**
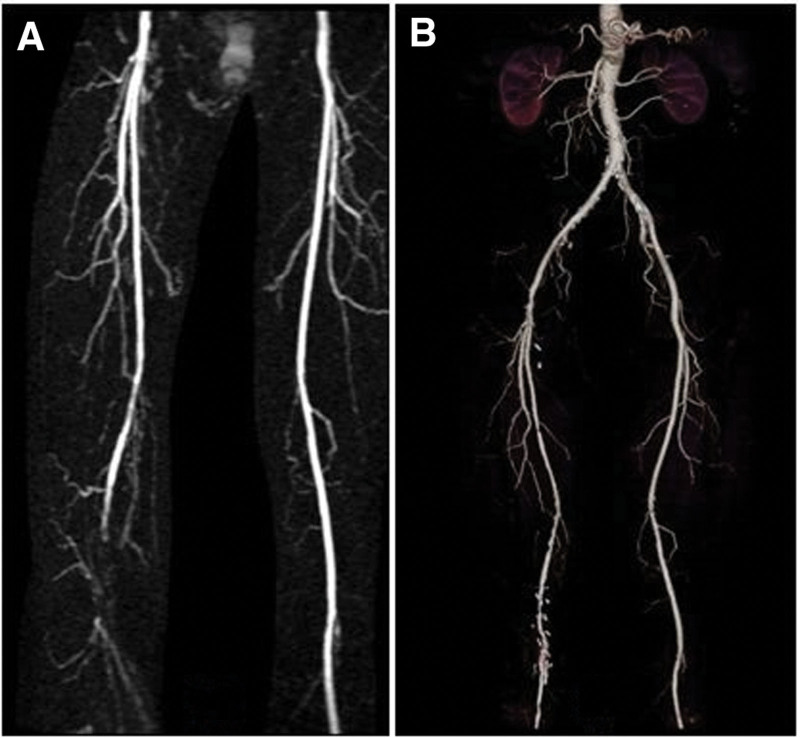
(A) Preoperative angiographic image. (B) Postoperative angiographic image.

Approval for this study was obtained from the Local Clinical Research Ethics Committee (No. 201/40). In this study, we examined retrospectively the results of 14 patients in total, who were admitted to our clinic with lower limb ischemia for whom thromboendarterectomy + patch plasty operation was applied using the posterior approach due to isolated popliteal artery occlusion. Patients who were symptomatic (Fontaine Stages 2b and 3) under maximal medical treatment, patients with short segmental lesions, and patients with 2/3 of the below-knee veins intact or with passage of contrast material on angiographic imaging were included in the study. Patients with lesions affecting other parts of the femoropopliteal segment and those who underwent different surgical methods were excluded from the study. Additionally, patients with thromboangiitis obliterans, popliteal artery entrapment syndrome, or embolism were not included in the study. The patients were assessed in terms of age, sex, and risk factors such as accompanying diseases and smoking, surgical method, anesthesia, incision type, preoperative and postoperative pulse examination, ankle-brachial index (ABI), wound infection, postoperative complications, treatment applied, and patency. All patients were diagnosed by physical examination before magnetic resonance imaging and/or computed tomography (CT) angiography (Fig. 1A, B). The patients were followed up on postoperative 10th day and at the 1st, 3rd, 6th, and 12th months. Physical examination, ABI measurement, and arterial and/or venous Doppler ultrasonography (USG) were performed as postoperative controls. Imaging was performed in patients using magnetic resonance and BT angiography, when necessary.

### 2.2. Surgical procedure

**Figure 2.**
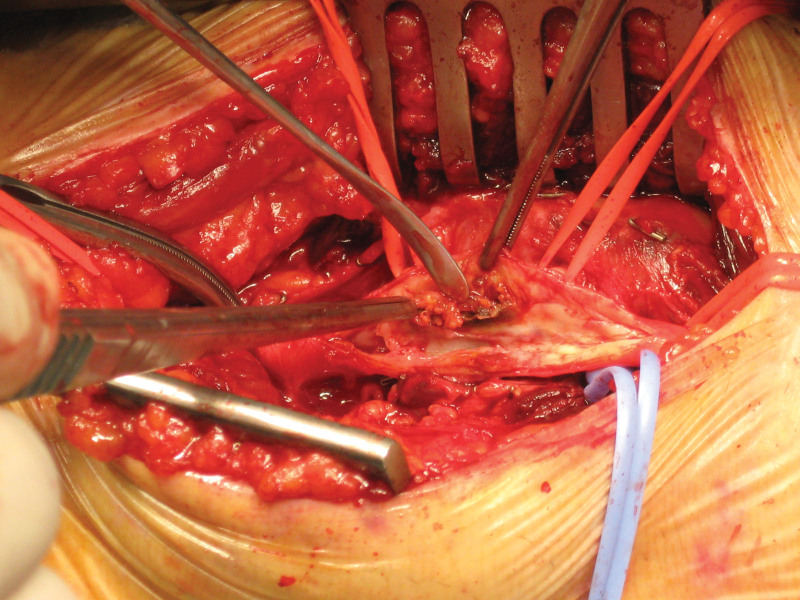
Endarterectomy procedure.

**Figure 3.**
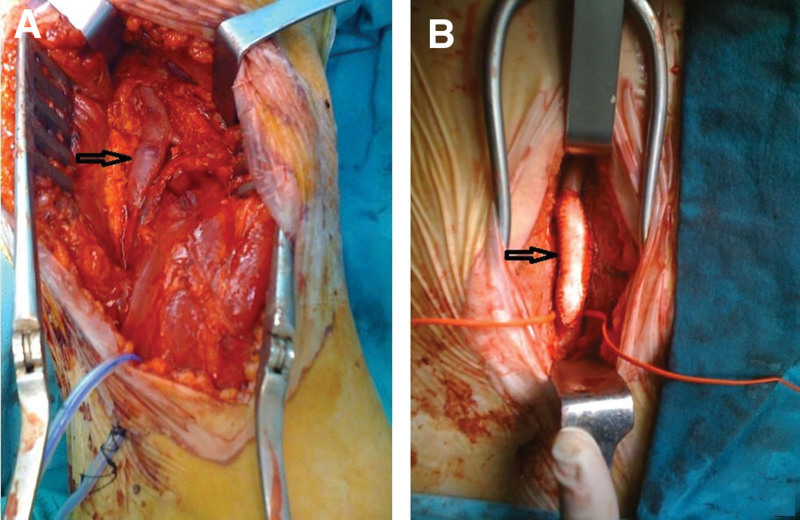
(A) Patch plasty with sort saphenous vein graft. (B) Patch plasty with PTFE graft. PTFE = polytetrafluoroethylene.

The patients were placed in the prone position following suitable anesthesia. A vertical or oblique “S” incision (medial to lateral) was made posterior to the popliteal fossa. The surgeon was allowed to select the incision type. The fascia was opened while taking precautions to avoid damage to the sural nerve and the small saphenous vein, and the tibial and common peroneal nerves were dissected. The popliteal artery was explored by contacting the popliteal vein as little as possible and was controlled with silastic loops. Furthermore, 5000 U of unfractionated heparin was administered intravenously before placing a clamp on the popliteal artery. The activated clotting time was maintained above 200 seconds. Vertical arteriotomy was performed after placing a vascular clamp. Endarterectomy was performed on the atherosclerotic and ulcerated plaque structures using a Watson Cheyne dissector with organized thrombus materials in the popliteal artery lumen (Fig. 2). Furthermore, thrombectomy was performed proximally and distally using 4F and 5F Fogarty catheters. When necessary, the intima was stabilized using 7/0 polypropylene sutures in the distal popliteal artery. The patch plasty procedure was applied to all patients with a continuous suturing technique using a 6/0 polypropylene suture, with a small saphenous vein graft or polytetrafluoroethylene (PTFE) graft prepared in suitable dimensions according to the surgeon’s preference (Fig. 3A,B). The operation was terminated by closing the tissues according to the plies following bleeding control and placement of a Hemovac drain. No other peripheral bypass procedures were performed in any patient in the study group.

Postoperative patients were administered continuous unfractionated heparin with an ACT target of 150 to 200 seconds for the 1st 24 hours, followed by intravenous 5000 U of unfractionated heparin 4 times a day, until they were discharged from the hospital. Antioxidant drugs were added to the treatment regimen on the 1st postoperative day. Patients with distal pulses that could be palpated during discharge were administered 300 mg acetylsalicylic acid + 75 mg clopidogrel once daily, and patients whose distal pulses could not be palpated were administered 100 mg cilostazol twice a day in addition to these treatments. Furthermore, statin treatment was administered to all the patients.

### 2.3. Statistical analysis

Statistical analysis was performed as a continuous variable (mean ± standard deviation). The categorical variables were given in the form of frequency and percentage.

## 3. Results

Twelve (85.7%) of the patients in our study were male, 2 (14.3%) were female, and the average age was 56.1 ± 9.9 (21–82) years. Nine (64.2%) patients had hypertension (HT), 7 (50%) patients smoked, and 4 (28.5%) patients had diabetes as a risk factor. Limb ischemia was severe in 11 patients (78.5%) patients (ABI < 0.7). All patients were at Fontaine Stages 2b and 3. Furthermore, 6 (42.8%) patients underwent surgery under general anesthesia, 5 (35.8%) under spinal anesthesia, and 3 (21.4%) under epidural anesthesia. Moreover, 8 (57.1%) patients had left and lower limb complaints and 6 (42.8%) had right lower limb complaints. The operation was performed in 9 (64.3%) patients with an oblique “S” incision and in 5 (35.7%) patients with a vertical incision. For patch application, a PTFE graft was used in 11 patients (78.6%), and a small saphenous vein graft material was used in 3 patients (21.4%). The patient data are provided in Table 1.

**Table 1:** Demographic and operative data.

	Patient number (n)	Percent
Sex		
Male	12	85.7
Female	2	14.3
Hypertension	9	64.2
Smoking	7	50
Diabetes	4	28.5
Anesthesia type		
Generally	6	42.8
Spinal	5	35.8
Epidural	3	21.4
Incision		
Oblique “S”	9	64.3
Vertical	5	35.7
Graft		
PTFE	11	78.6
Saphaneous vein	3	21.4

PTFE = polytetrafluoroethylene.

During the postoperative examination of the patients, while the distal pulse of 9 patients (64.3%) could be palpated, there was a strong Doppler flow effect in distal arteries in 5 (35.7%) patients. Evident decrease was observed in the limb ischemia of the patients, and an increase was observed in their early and mid-term ABIs and walking distances. The preoperative and postoperative ABI changes of the patients are presented in Table 2. The data of the patients who were lost during follow-up after the postoperative 6th month are not indicated in Table 2.

**Table 2:** Ankle-brachial index change.

	ABI				Average ABI
	0.7>		0.7<		
	Patient number (n)	Percent	Patient number (n)	Percent	
Preoperative	11	78.5	3	21.5	0.6
Postoperative	6	43	8	57	0.85
1 mo	2	14	12	86	0.97
3 mo	1	8	13	92	0.98
6 mo	1	8	13	92	1.02

ABI = ankle-brachial index.

The postoperative hospitalization period was 8 ± 3.7 (4–14) days on average. The average length of the follow-up of the patients was 17 ± 3.4 (9–33) months. No thrombosis or any related complication that required secondary intervention developed in any of our patients in the postoperative early period. No wound infection was encountered in any patient. Again, it was observed that almost all of the patients who underwent a vertical incision had complaints, such as difficulty in bringing the leg to full extension, tension, and pain during mobilization in the early period. Tibial nerve damage-related complaints proved with electromyography occurred in 1 patient (14.2%). A 2 × 4 cm hematoma was detected in the popliteal fossa in the postoperative 2nd week in another patient (14.2%). However, no surgical intervention was performed since it did not push the vascular structures, and it was observed that it regressed in the follow-ups. Furthermore, no deep vein thrombosis related to popliteal vein manipulation was encountered in any patient. No amputation was applied to any of the patients in the early period, and no significant morbidity and mortality were observed in the postoperative early period.

While our patency rate in the first 6 months of the postoperative late follow-up period was 100%, it was 92.8% in the 1st year and 85.7% in the 2nd year. In the 1st-year control group, 1 patient complained of claudication that had started shortly before. The popliteal artery was thrombosed and treated with a femoral thrombectomy. In another patient, thrombosis was observed in the popliteal artery on Doppler USG performed 3 years later as a result of not taking the popliteal pulse. The patient was followed up medically, without performing any surgical or interventional procedures, as did not have any complaints and was asymptomatic, probably because of the developed collateral vascular structure. Above-knee amputation was performed in 1 patient, who delayed their regular follow-ups after the 6th month, applied to our clinic in the late period, and did not have a good below-knee vascular bed 2 years later. Another patient underwent the 1st below-knee bypass with the reverse great saphenous vein 1.5 years later, followed by the below-knee amputation procedure 2 years after this procedure. One of our patients, who did not have any vascular complaints during the follow-up period, was lost due to malignant melanoma 3 years later, and the other were lost due to cardiac reasons 2 years later.

## 4. Discussion

Circulation disorders that develop as a result of PADs are an important cause of morbidity and mortality, and they significantly affect the quality of life of patients owing to inadequate circulation of the extremities.^[3]^ Popliteal artery occlusion is generally the final stage of the atheromatous plaque formation. These lesions may occur as stenoses or as complete obstructions of the popliteal artery. In several studies, smoking was positively correlated with HT, hypercholesterolemia, and diabetic PAD.^[4,5]^ It was observed that the patients in our study also had similar risk factors, such as HT in 9 patients (64.2%), smoking in 7 patients (50%), and diabetes in 4 patients (28.5%).

The clinical status of patients with popliteal artery occlusion is variable, and patients can have intermittent claudication, critical ischemia, or acute thromboembolism, depending on whether there is any accompanying pathology in other vascular segments according to the level of involvement in the artery.^[6]^ The complaints at presentation in our patients were mainly intermittent claudication and resting pain. Limb ischemia was serious in 11 (78.5%) patients at Fontaine Stages 2b and 3, and the ABI was <0.7.

The treatment was shaped according to the clinical status of the patient and radiological imaging findings. The best method for eliminating the symptoms of atherosclerotic occlusive lesions or saving the limb is to find an open artery in the distal region and to ensure flow again. According to the Trans-Atlantic Inter-Society Consensus II report, lesions in the lower extremity arteries are classified, and surgical interventions are preferred for Type D lesions.^[7]^ Regardless of the reason for popliteal artery occlusion, endovascular and/or surgical intervention is necessary in patients with severe claudication that changes their life and does not respond to medical treatment, and in patients with critical limb ischemia.^[7]^

When the popliteal artery is compared to femoral vascular structures, it is characterized by different embryologic and anatomic features, and it takes its roots from the sciatic system embryologically. Furthermore, the popliteal artery is known to stay between muscle groups when compared to the femoral artery, and it is known to be under biomechanical stress resulting from many torsional movements, such as flexion and extension, that affect the knee.^[8,9]^ High biomechanical stress in the region due to this anatomic structure negatively affects patency rates related to popliteal artery bypass procedures. This biomechanical stress causes a high rate of stent fracture and occlusion, accelerating restenosis.^[10,11]^ In studies conducted, while percutaneous transluminal angioplasty results are good in popliteal artery stenoses in which the distal flow is sufficient, the results are far from satisfactory in intense calcific, occlusive lesions in which the distal flow is bad.^[12,13]^ Furthermore, neointimal hyperplasia tended to increase at this location. Newly developed biodegradable stents have been used with drug-coated and bare stents in these types of lesions; however, there is no consensus on their efficacy and they remain controversial.^[14]^

Among surgical options, bypass surgeries performed at various levels using different graft materials, such as autologous, synthetic (Dacron and PTFE), and biological grafts, are widely implemented. Performing bypass surgery on the popliteal and infrapopliteal arteries is crucial in vascular surgery. It has been shown that the best method for improving patient symptoms or saving patient limbs is to restore distal artery flow.^[15]^ This is ensured by performing a bypass procedure on the popliteal, anterior tibial, tibioperoneal trunk, posterior tibial, peroneal, or plantar arteries. The autologous saphenous vein is the preferred graft material in below-knee femoropopliteal bypass applications and is superior to synthetic grafts in terms of short- and long-term outcomes.^[16]^ The advantages of the saphenous vein graft include its resistance to infections, the fact that it is less thrombogenic, its suitability for manipulation, its ability to maintain its vitality for a long time, the fact that it is fed by diffusion, and its ease of procurement.^[17,18]^ While PTFE grafts are an alternative to autogenous saphenous grafts, the remaining open ratios of these grafts are low compared to those of the saphenous vein.^[19]^ Moreover, biosynthetic vascular grafts are widely preferred for below-knee bypass.

All of these techniques are used in lesions that are considered to be of higher priority, as they keep the femoropopliteal artery segment diffused or segmental. In this study, we aimed to postpone the below-knee bypass option by applying this technique only in patients with isolated popliteal artery stenosis. Furthermore, the fact that the majority of these patients are potential candidates for coronary artery bypass surgery is of particular importance, and care must be taken to protect the great saphenous vein. Many studies have demonstrated that popliteal endarterectomy is advantageous in terms of protecting the great saphenous vein, which allows the use of above- or below-knee femoropopliteal bypass in subsequent coronary artery bypass surgery.^[20]^

The patch plasty procedure was applied with a short graft in patients with isolated popliteal artery occlusion after performing popliteal artery endarterectomy using the posterior approach. The advantages of this technique include the use of a short incision, the fact that atherosclerotic structures are totally removed with endarterectomy, ease of access to trifurcation arteries, and the unused great saphenous vein. We preferred to use a PTFE graft first, rather than a saphenous vein graft, in most patients for patch plasty. While we used the PTFE graft in 11 patients (78.6%), we used a piece of the graft from the small saphenous vein because the lesion was short in 3 patients (21.4%). Studies have indicated that the incidence of aneurysmal degeneration and intimal hyperplasia development is lower in PTFE-graft use when compared to the use of the saphenous vein graft.^[21]^ Dacron graft materials have been used in several studies.^[22]^ In addition, in some suitable lesions, the eversion popliteal endarterectomy technique can also be applied.^[23]^

The success rate of the procedure was 100% during short-term postoperative follow-up. No thrombosis or complications that required secondary intervention developed in any of the patients during the early follow-up period. A 2 × 4-cm hematoma was detected in the popliteal fossa in the 2nd week of USG in only 1 patient. However, no surgical intervention was performed because it did not push the vascular structures, and they were resorbed and regressed during follow-up. Although no postoperative wound infection was observed, wound healing lasted longer for patients in whom a vertical incision was made. Moreover, it was observed that almost all patients in whom the vertical incision was applied had complaints such as difficulty in bringing the leg to full extension, tension, and pain during mobilization. We believe that our first preference must be in the form of an “S” incision to minimize such negativities in the approach used to treat this type of lesion.

Peripheral nerve damage is generally observed during exploration of popliteal aneurysms. However, temporary complaints related to tibial nerve damage, the diagnosis of which was confirmed with electromyography, occurred in 1 (14.2%). This was addressed using medical treatment. Vein damage related to popliteal vein manipulation and deep vein thrombosis were not encountered in any of the postoperative patients. Another concern is the limb amputation. The biggest risk factor for amputation is the presence of diabetes.^[24]^ Although 4 (28.5%) of our patients had diabetes, none of them underwent amputation in the early postoperative period; however, it was necessary to perform amputation in 2 patients in the long term.

In a study in which popliteal endarterectomy was performed, the frequencies in the 1st, 6th, 1st, and 3rd year were 97.8%, 95.7%, 93.6%, and 89.3%, respectively.^[24]^ In addition, in the study conducted by Gaudin et al^[23]^ in recent years, they reported that they were 100% technically successful during their average 3-year follow-up and that the patency rate after the procedure was high. In our study, while the patency rate in the first 6 months of postoperative follow-up was 100%, it was 92.8% in the 1st year and 85.7% in the 2nd year. In their study, Nasr et al^[2]^ demonstrated an increase in the postoperative walking distance of patients, whereas regression was observed in ischemic pain, and their symptoms improved. In our cases, the pulses were palpated in 9 (64.3%) patients during the postoperative pulse examinations. While the pulse could not be palpated, there was a strong Doppler flow effect in the distal arteries in 5 (35.7%) patients. Again, a significant increase in walking distance and a significant decrease in limb ischemia were observed in these patients, and their rest of the pain was eliminated. Moreover, there was an increase of almost twice the average ABIs in the preoperative and postoperative 1st, 3rd, and 6th months: 0.6, 0.85, 0.97, 0.98, and 1.02, respectively.

There is no doubt that one of the most crucial factors affecting the success of the procedure performed is the presence of distal vascular bed disease. If the distal vascular bed is significantly affected by atherosclerosis, the long-term success of the procedure is low. Regardless of the preferred treatment method for PADs, they cannot be completely treated because atherosclerotic diseases tend to progress. Although endovascular procedures have gained importance in the treatment of patients requiring revascularization in recent years, conventional surgery remains the gold standard treatment. Isolated popliteal artery diseases are pathologies that can be diagnosed quickly and correctly using radiological methods, but they are sometimes difficult to diagnose clinically. The results of surgical treatment can be limb-saving when diagnosed correctly and quickly.

This study had some limitations. First, the number of patients was small because it was difficult to identify a patient with isolated popliteal artery occlusion. Second, there has been no comparative study using another technique. In fact, rather than for comparison, our main purpose was to emphasize that the method we applied is still valid and can be applied safely by vascular surgeons in cases where other methods are not suitable, fail, or cannot be reached.

## 5. Conclusion

Surgical treatment with the posterior approach in isolated popliteal artery lesions has low perioperative morbidity and mortality rates, and sufficient recanalization rates. Treatment methods that are alternatives to open surgical procedures may also be considered in high-risk patient groups with claudication and/or critical leg ischemia accompanied by comorbid factors. Furthermore, we believe that this method can be applied safely by vascular surgeons because it allows below-knee femoropopliteal bypass, which is the subsequent stage of treatment, and because the great saphenous vein is protected, as well as because of its acceptable early and mid-term results.

## Author contributions

**Conceptualization:** Serhat Huseyin.

**Data curation:** Serhat Huseyin, Orkut Guclu, Volkan Yuksel, Selami Gurkan.

**Formal analysis:** Serhat Huseyin, Adem Reyhancan.

**Methodology:** Serhat Huseyin, Suat Canbaz.

**Project administration:** Serhat Huseyin, Orkut Guclu.

**Resources:** Serhat Huseyin, Orkut Guclu, Adem Reyhancan.

**Supervision:** Volkan Yuksel, Suat Canbaz.

**Validation:** Serhat Huseyin, Selami Gurkan, Suat Canbaz.

**Visualization:** Serhat Huseyin, Orkut Guclu, Adem Reyhancan.

**Writing – original draft:** Serhat Huseyin.

**Writing – review & editing:** Serhat Huseyin, Orkut Guclu.
